# Non-intubated Thoracic Surgery: Wedge Resections for Peripheral Pulmonary Nodules

**DOI:** 10.3389/fsurg.2022.853643

**Published:** 2022-04-07

**Authors:** Vincenzo Ambrogi, Alexandro Patirelis, Riccardo Tajè

**Affiliations:** Thoracic Surgery Department, Tor Vergata University Policlinic of Rome, Rome, Italy

**Keywords:** lung cancer, wedge resection, non-intubated anesthesia, VATS, sublobar lung resection

## Abstract

The feasibility of performing pulmonary resections of peripheral lung nodules has been one of the main objectives of non-intubated thoracic surgery. The aim was to obtain histological characterization and extend a radical intended treatment to oncological patients unfit for general anesthesia or anatomic pulmonary resections. There is mounting evidence for the role of wedge resection in early-stage lung cancer treatment, especially for frail patients unfit for general anesthesia and anatomic resections with nodules, demonstrating a non-aggressive biological behavior. General anesthesia with single lung ventilation has been associated with a higher risk of ventilator-induced barotrauma and volotrauma as well as atelectasis in both the dependent and non-dependent lungs. Nonetheless, general anesthesia has been shown to impair the host immune system, eventually favoring both tumoral relapses and post-operative complications. Thus, non-intubated wedge resection seems to definitely balance tolerability with oncological radicality in highly selected patients. Nonetheless, differently from other non-surgical techniques, non-intubated wedge resection allows for histological characterization and possible oncological targeted treatment. For these reasons, non-intubated wedge resection is a fundamental skill in the core training of a thoracic surgeon. Main indications, surgical tips, and post-operative management strategies are hereafter presented. Non-intubated wedge resection is one of the new frontiers in minimal invasive management of patients with lung cancer and may become a standard in the armamentarium of a thoracic surgeon. Appropriate patient selection and VATS expertise are crucial to obtaining good results.

## Introduction

Since the very beginning of the non-intubated thoracic surgery program, the feasibility of pulmonary resections of peripheral lung nodules has been one of the main objectives (1). The aim was to obtain histological characterization and extend a radical intended treatment to oncological patients unfit for general anesthesia or anatomic pulmonary resections. Growing pieces of evidence define the role of wedge resection in early-stage lung cancer treatment for nodules demonstrating a non-aggressive biological behavior, especially for frail patients unfit for anatomic resections (2). In particular, wedge resection demonstrated a higher overall 5-year survival and a lower recurrence rate compared to non-surgical strategies (3–5). General anesthesia with single lung ventilation has been associated with a higher risk of ventilatory-induced barotrauma and volotrauma as well as atelectasis in both the dependent and non-dependent lungs (6). Nonetheless, general anesthesia has been shown to impair the host immune system, eventually favoring both tumoral relapses and post-operative complications (7). Thus, non-intubated wedge resection seems to definitely balance tolerability with oncological radicality in highly selected patients. Main indications, surgical tips, and post-operative management strategies are, hereafter, presented.

## Patient Selection

First of all, general indications for wedge resection, as outlined in Table 1, should be verified. Nonetheless, frail patients unfit for an anatomic resection, presenting with more aggressive nodules, may be discussed in a multidisciplinary setting, including pulmonologists, oncologists, radiotherapists, radiologists, and thoracic surgeons in order to assess the functional reserve of the patients and to ensure the best clinical management. If the patient is fit for chemo or targeted therapies, surgical biopsy gives an adequate specimen for molecular characterization, allowing further medical-targeted therapies. Nonetheless, the non-intubated surgical biopsy, differently from computed tomography core needle biopsy, allows lymph node sampling, possibly affecting the treatment choice (8). In patients with low performance status, optimal surgical treatment is not allowed. In these patients, non-intubated wedge resection offers in the same minimally invasive procedure both a certain diagnosis as well as a definitive treatment.

**Table 1:** General indication for wedge resection.

- Poor pulmonary reserve or other comorbidities contraindicating anatomical resections and/or - Nodule located in the outer third of the lung (peripheral) ≤ 20 mm with at least one of the above characteristics of a non-aggressive behavior: - Pure adenocarcinoma *in situ* histology - ≥50% of the nodule has a ground-glass appearance - Doubling time ≥400 days at radiological follow-up
For all the nodules, a 20 mm margin (or a margin greater or equal to nodule diameter) macroscopical free from tumoral cells should be guarantee

Attention should be paid to general indications of video-assisted thoracic surgery (VATS). A clinical history at high risk of extensive pleural adhesions may hinder the thoracoscopic procedure. Successively, indications of non-intubated wedge resection should be verified (9). Particularly, a rapid resection with at least a 2-cm resection margin free of a tumor should be guaranteed. According to current guidelines, a parenchymal resection margin greater or equal to a nodule diameter is also considered adequate (2). Under this perspective, a nodule diameter and localization are crucial. As a general rule, the smaller the nodule, the easier the resection. In our experience, the cut-off may be set to 2 cm, but resection of larger nodules may be attempted. Localization of the nodule is the other key aspect. The nodule should be located peripherally, defined as the outer third of the lung, to allow quick nodule detection and resection, avoiding damages of main pulmonary vascular branches. Thus, the distance between the nodule and the visceral pleura may be shorter than the nodule diameter. In most of the cases, a length of 1 cm from the visceral pleura to the nodule is acceptable. Noticeably, an emphysematous pliable lung under spontaneous ventilation may help resect deeply located nodules—otherwise, unresectable. Nodules located deeper in the parenchyma may undergo resection if they are in proximity with the fissure but still far from the main vascular structures. Nodules located at the bases of the inferior lobe may be of concern due to the proximity with the inferior pulmonary vein. In these cases, as a first step, the pulmonary ligament should be sectioned in order to mobilize the inferior lobe and identify the inferior pulmonary vein. An accurate three-dimensional radiological planning should be made based on axial, sagittal, and coronal scans of a pre-operative computed tomography to localize the nodule. Finally, patient selection should also consider patient will and behavior. A very anxious patient may not tolerate the procedure. Defining contraindication to non-intubated wedge resection can be complex. The different components of the procedure should be carefully and singularly evaluated, and the best strategy should be tailored to patient characteristics. The main contraindications for the non-intubated wedge resection include both contraindications for non-intubated anesthesia and contraindications for thoracoscopic wedge resection as listed in Table 2 (10, 11).

**Table 2:** General contraindication to non-intubated wedge resection for peripheral pulmonary nodules.

- Contraindication to VATS - Pre-operative high suspicious of diffuse pleural adhesions - Previous homolateral thoracic surgery (relative contraindication) - Bleeding disorders or abnormal coagulation tests - Obesity (Body Mass Index ≥ 30 Kg/m^2^) (discussed) - Contraindication to wedge resection - Tumoral diameter >30 mm - Feasibility of anatomic resections - Nodule distance from the visceral pleural >10 mm or in proximity to main vascular structures - Contraindication to non-intubated wedge resection anesthesia - Feasibility of general anesthesia with single lung ventilation - Estimated difficult orotracheal intubation - Unstable or anxious psychological profile - American Society of Anesthesiologists Physical Status Classification ≥ III - Obesity (Body Mass Index ≥ 30 kg/m^2^) - Contraindication to TEA - High risk of intraoperative seizure - Brain edema - Spine deformities - Bleeding disorders - Hemodynamic instability - Contraindication to local anesthesia - Previous allergy to local anesthesia - Estimated long surgical duration

*TEA, thoracic epidural anesthesia; VATS, video-assisted thoracic surgery*.

## Anesthesia

The non-intubated program takes advances both from innovation in surgery and in anesthetic techniques. From triportalthoracoscopic access under thoracic epidural anesthesia (TEA) to uniportal thoracoscopic access under monitored-based anesthesia, surgical and anesthesiologic sciences have been integrated to reduce surgical invasiveness (1). In non-intubated surgery, spontaneous breathing and diaphragmatic contraction are preserved. As a consequence, the cough reflex is not inhibited and may hinder a surgical procedure. This physiologic side effect may be negligible in wedge resection because peripheral pulmonary manipulation only scarcely triggers the cough reflex. Whether a better control of cough reflex is mandatory for the surgical procedure, atropine (0.01 mg/kg) such as a vagal block (12), 5 ml of aerosolized 2% lidocaine or other techniques can be used (13, 14). Hereafter, the main characteristics of the different anesthesiologic techniques are presented.

### Thoracic Epidural Anesthesia

TEA was the first anesthesiologic approach to non-intubated surgery. The mixture of a local anesthetic composed of 1.66-mg/ml sufentanil and 0.5% ropivacaine is continuously released through an epidural catheter placed at the fourth thoracic nerve level. This anesthesiologic strategy simultaneously covers three or four metameric regions, allowing multiport access (15). If the analgesic effect is unsatisfactory, the somatosensory block may be implemented with a supplementary injection of 7.5% ropivacaine and 2% bupivacaine at incision sites (16). Previous reports have demonstrated that TEA may have a higher complication burden when compared with other central neuroaxial blocks and may hinder the heart response to hemodynamic challenges in selected patients (17, 18). Nonetheless, the progressive reduction in the number of surgical ports, from three to single surgical access, allowed the transition toward a less-invasive anesthesiologic strategy.

### Local Anesthesia

The accomplishment of wedge resection through two or even one port allowed the transition from TEA toward local anesthesia with intercostal blockage. To achieve suitable pain control, a mixture of short- and long-acting local anesthetics may be used, infiltrating the different layers of the surgical access. Particularly, lidocaine, 2% (4 mg/kg), can be used at the site of the surgical incision to ensure rapid analgesia of the trajectory of the surgical incision up to the parietal pleura. Successive direct infiltration of the competent intercostal nerve with 7.5% ropivacaine (2 mg/kg) helps to empower the somatosensorial blockage and to ensure analgesia throughout the procedure. Paravertebral block (PVB) may be used as an alternative to TEA. A local anesthetic, a mixture of ropivacaine, is delivered under ultrasonographic guidance in the paravertebral region. Eventually, a catheter can be positioned, allowing post-operative local anesthetic infusion. PVB has similar antalgic effects and a lower complication rate when compared with TEA (19). The risk of iatrogenic pneumothorax is negligible as a thoracic procedure with chest tube positioning is planned. Other procedures such as a serratus anterior plane block can be used as a supplementary pain relief strategy (20).

### Monitored Anesthesia

At the beginning of the non-intubated program, all the procedures were performed in completely awake patients. Nowadays, awake thoracoscopic surgery is only one of the different procedures that may be performed under non-intubated anesthesia. The introduction of new technologies such as the Bispectral Index (BIS) allowed for operating on mildly sedated patients. Particularly, BIS helps to monitor the consciousness state of the patient even during the procedure and eventually adjust drug dosage and Delivery in Order to Keep the Patient Awake (BIS Index 90–100) or to Obtain Mild Sedation (BIS 60–90) in anxious patients (21, 22). In pulmonary nodules, wedge resection operating on completely awake patients may be disadvantageous. Patients undergoing this procedure add to the surgery-related fear, the anxiety of a probable cancer diagnosis. This supplementary emotional stress may be implemented during the surgical procedure, leading to panic attacks or discomfort. Thus, cautious use of mild sedation may help to relieve this tension. Nonetheless, deeper sedation can be useful during the demanding part of the procedures. In these instances, intravenous propofol (10 mg/ml) with an increasing dosage of fentanil may be used.

## Surgical Techniques

### Surgical Access

#### Triportal VATS

At the beginning of the non-intubated program, the triportal VATS approach was the preferred approach to peripheral pulmonary nodules wedge resection (16). With the experience made in hundreds of non-intubated procedures, at our institution, this approach has been gradually put aside. However, this access may be particularly fit for beginners, allowing extensive lung manipulation in order to reduce the movements of a ventilated lung (21). Due to the three different surgical access, a TEA anesthetic approach seems to be the most suitable, covering simultaneously several intercostal spaces, but multiple site injections of local anesthetic can be also performed. As in classical triportal VATS, the ports are placed in a so-called baseball-diamond setting (9).

#### Biportal VATS

As in VATS performed under general anesthesia, the number of access sites has been reduced, avoiding the need of the second operative port. This reduction in the number of surgical access sites both reduces post-operative pain and allowed the transition from TEA to monitored-based local anesthesia (1, 23). Surgical access position does not significantly differ from triportal access. The annular sleeve-shaped self-retractor for thoracoscopic surgery can enlarge the operative access, allowing palpation, while minimizing surgical trauma on the intercostal nerve. To provide the best visualization and to ensure a consistent tumor-free margin, the position of the camera and the stapler can be interchanged.

#### Uniportal VATS

With the introduction of uniportal VATS under general anesthesia, this technique has rapidly been implemented in the non-intubated program (1). Uniportal VATS has been demonstrated to reduce pain, nerve injury and paresthesia, and length of hospital stays compared with classical multiport VATS (23). An access point 3 cm in length is placed at the fifth intercostal space along the anterior axillary line. Due to the single port, strategies to reduce respiratory movements and instruments conflict should be implemented such as the intraoperative vagal block or anesthesiologic strategies to reduce mediastinal oscillation. The intraoperative vagal block, obtained by injection of 3 ml of bupivacaine, has been implemented in order to reduce respiratory movements, allowing better visualization of the lung. The suitable anatomical landmarks to identify this structure are the lower paratracheal and the aortopulmonary window for right and left side procedures, respectively (24). When a laryngeal mask is used, mediastinal oscillation can be reduced, adjusting breathing frequency and tidal volume or delivering a supplementary dosage of remifentanil (0.04 mg/kg/min). Cautious ventilatory parameters and drugs dosage titration are necessary to avoid acid-based imbalance and severe hypercapnia (25). A sleeve-shaped retractor and specific uniportal-designed instruments reduce conflict and ameliorate visualization (21).

### Nodule Detection and Resection

Since the time factor is crucial in non-intubated surgery, strategies to quickly detect nodules deserve particular attention. Pre-operatively, a surgical and radiological plan with a clear three-dimensional localization of the nodule is fundamental, and radiological imaging should be available in the operative room at any time during the procedure. If the nodule is deemed to be difficult to detect intraoperatively, pre-operative needle localization can be achieved. Intraoperatively, visualization of visceral pleural retraction or scarring can be the landmark of an underlying neoplastic process. Nonetheless, instrument palpation can be performed by sliding a ring forceps above the lung parenchyma. An impaired consistency would determine a glimpse in the sliding forceps, revealing the nodule. Other techniques such as intraoperative ultrasound or dye tracers have been applied to detect pulmonary nodules with different results (26, 27). Nonetheless, in the author's experience, direct digital palpation through the operative port is the best and fastest strategy to detect the nodule. Before the procedure, a time cut-off for the detection of the nodule should be determined. If the detection fails, do not hesitate to enlarge the incision. Obviously, a supplementary local anesthetic injection can be necessary to preserve analgesia. When the nodule has been finally detected, a vascular clamp can be placed under the nodule, facilitating the stapler bite positioning. In case of uniportal VATS, to further reduce the number of surgical instruments occupying the single-port access, one or more 2–0 silk sutures adjacent to the nodule can help to elevate it, facilitating resection. Resection can be accomplished by mechanic staplers. The surgeon can choose between short and long bite staplers. The former can be easily moved inside the pleural cavity with the lung partially deflated and allow for the description of a curvilinear pathway necessary to preserve the 2 cm free from a tumor margin. The latter allows a faster resection with less firing, but intrapleural movement may be difficult. The resected parenchyma including the nodule should be included in a retrieval bag before taking it out from the thorax. Hemostasis and pneumostasis should be checked, and a single chest drain tube can be positioned toward the apex of the pleural cavity under thoracoscopic vision. In uniportal VATS, a single tube is positioned in the middle thoracic single incision, and it is directed toward the basis and the pleural apex in a J shape. As tube dislocation, after pulmonary re-expansion is frequent, possible post-operative pleural effusion may be inadequately drained. The liquid accumulating at the base of the pleural cavity may hinder lung complete re-expansion, favoring post-operative infections. Therefore, a T-shaped tube with two extremities directed toward both the apex and the basis of the pleural cavity has been recently implemented at our institution (28).

## Intraoperative Complications and Shift To General Anesthesia and/or Conversion To Open Surgery

The management of intraoperative complications may require orotracheal intubation and conversion to general anesthesia (29). In non-intubated wedge resection, anesthesiologic complications do not significantly differ from the ones characterizing other non-intubated procedures. These complications may require conversion to general anesthesia with or without conversion to thoracotomy. Hypercapnia leading to acidosis and hypoxemia is the most relevant (30). Hypercapnia is generally self-limiting. The so-called permissive hypercapnia follows the rebreathing phenomenon. Avoiding selective lung ventilation leads to air interexchange between lungs during surgical pneumothorax. However, this alteration is usually well-tolerated and goes to resolution at the end of surgery (22). Whether hypercapnia does not resolve or hypoxemia develops, high-flow oxygen masks or, if oxygen saturation is <90%, non-invasive Bilevel Positive Airway Pressure can be used, facilitating lung recruitment and adjusting a ventilation-to-perfusion mismatch. Difficulties in a nodule finding can follow insufficient deflation of the lung, unbearable cough, or extensive pleuro-parenchymal adherence. In the case of insufficient deflation of the parenchyma, additional small surgical access allows the introduction of a surgical instrument to retract the lung. If the patient is ventilated through a supra-glottic device, such as a laryngeal mask, ventilatory strategies to regularize breathing pulmonary movement may be applied, facilitating the resection. Strategies to control cough reflex have already been discussed. Energy devices can control pleuro-parenchymal adhesion as in conventional VATS. As afore-mentioned, it is pivotal to decide before the procedures a time cut-off before enlargement of the surgical access or conversion to general anesthesia.

Hemorrhages management depends mostly on the surgeon's thoracoscopic ability and can follow usual thoracoscopic guidance (31). It is remarkable that wedge resections rarely result in massive bleeding. If hemorrhages happen, a non-intubated thoracoscopic approach can be maintained if the surgeon can easily control the bleeding and the hemodynamic is deemed stable. Otherwise, urgent conversion in general anesthesia with or without thoracotomy is necessary.

Non-intubated thoracoscopic anatomic resections are technically demanding and require proficiency in thoracoscopic surgery, non-intubated surgery, and cardio-thoracic anesthesia. Nonetheless, in patients selected for non-intubated surgery, the pre-operative pulmonary function has to be carefully evaluated. Incautious anatomic resections in patients with marginal pulmonary function can be detrimental eventually leading to patient perioperative mortality. Therefore, at the beginning of the procedure, attention should be dedicated to assessing the resectability of the disease through a non-anatomic lung-sparing approach before any unreversible action.

As a general rule, conversion in general anesthesia with or without thoracotomy should never be considered as a failure but as a responsible decision for a patient's sake. Intubation and general anesthesia induction can be performed both in lateral decubitus or turning the patient in the supine position. In the latter option, a thoracic drainage device can be inserted in the chest cavity and one silk two stitch or surgical drapes can cover the surgical access to maintain sterility. During the change to supine position, the anesthesiologist becomes the team leader and controls head and neck (22).

## Post-Operative Management

Post-operative management of non-intubated wedge resection differs from the classical management of wedge resection performed under general anesthesia. In order to obtain the best results from the non-intubated technique, particular attention should be paid in strict adherent to enhanced recovery after surgery protocols (32). Patients can be early mobilized, and food and beverage oral intake can be restarted in the first hours from the procedure, thus avoiding intravenous fluids administration. Ambulation is strongly encouraged a few hours from the procedure. Pain control is achieved through oral analgesics and eventual intravenous moderate opioid-based analgesia (22, 33). A chest x-ray is performed between 2 and 8 h from the procedures, and chest drainage is removed as soon as no air leaks are detected and pleural collection is <200 ml in the day (24). Electronic drainage systems can be used. If air leaks persist longer than 6 days, dismission with a Heimlich valve can be considered. Comparing non-intubated VATS with VATS performed under general anesthesia, the former is associated with less post-operative complications and pain burden and a shorter hospital stay (34).

## Conclusion

With the increase in life expectancy and the wide diffusion of high-resolution radiological imaging, pulmonary nodules are becoming a quite frequent occurrence. Growing pieces of evidence are supporting non-intubated thoracic surgery in different settings. In a recent randomized trial, non-intubated VATS demonstrated to be non-inferior in the treatment of spontaneous pneumothorax. Non-intubated approaches showed better results also in thymectomy, lung volume reduction surgery, interstitial lung disease, and several other procedures. Non-intubated wedge resection is one of the new frontiers in minimal invasive management of patients with lung cancer and may become a standard in the armamentarium of a thoracic surgeon. Appropriate patient selection and VATS expertise are crucial to obtaining good results.

## Author Contributions

VA: critical and scientific review of the manuscript. RT: writing the manuscript. AP: review of the literature. All authors contributed to the article and approved the submitted version.

## Funding

This work was supported by post-graduate course funding.

## Conflict of Interest

The authors declare that the research was conducted in the absence of any commercial or financial relationships that could be construed as a potential conflict of interest.

## Publisher's Note

All claims expressed in this article are solely those of the authors and do not necessarily represent those of their affiliated organizations, or those of the publisher, the editors and the reviewers. Any product that may be evaluated in this article, or claim that may be made by its manufacturer, is not guaranteed or endorsed by the publisher.
